# Congenital anomalies and genetic disorders in neonates and infants: a single-center observational cohort study

**DOI:** 10.1007/s00431-021-04213-w

**Published:** 2021-08-04

**Authors:** A. Marouane, R. A. C. M. Olde Keizer, G. W. J. Frederix, L. E. L. M. Vissers, W. P. de Boode, W. A. G. van Zelst-Stams

**Affiliations:** 1 Department of Human Genetics, Radboud University Medical Center, Radboud Institute of Health Sciences, Nijmegen, The Netherlands; 2 Department of Health Sciences and Primary Care, University Medical Center, Utrecht, The Netherlands; 3 Department of Human Genetics, Donders Institute for Brain, Cognition and Behaviour, Radboud University Medical Center, Nijmegen, The Netherlands; 4 Department of Neonatology, Radboudumc Amalia Children’s Hospital, Radboud Institute of Health Sciences, Nijmegen, the Netherlands

**Keywords:** Diagnostic yield, Genetic diagnosis, Neonates, NICU, Neonatal intensive care unit

## Abstract

Neonates with genetic disorders or congenital anomalies (CA) contribute considerably to morbidity and mortality in neonatal intensive care units (NICUs). The objective of this study is to study the prevalence of genetic disorders in an academic level IV NICU. We retrospective collected and analyzed both clinical and genetic data of all 1444 infants admitted to the NICU of the Radboudumc (October 2013 to October 2015). Data were collected until infants reached at least 2 years of age. A total of 13% (194/1444) of the patients were genetically tested, and 32% (461/1444) had a CA. A total of 37% (72/194) had a laboratory-confirmed genetic diagnosis. In 53%, the diagnosis was made post-neonatally (median age = 209 days) using assays including exome sequencing. Exactly 63% (291/461) of the patients with CA, however, never received genetic testing, despite being clinically similar those who did.

*Conclusions*: Genetic disorders were suspected in 13% of the cohort, but only confirmed in 5%. Most received their genetic diagnosis in the post-neonatal period. Extrapolation of the diagnostic yield suggests that up to 6% of our cohort may have remained genetically undiagnosed. Our data show the need to improve genetic care in the NICU for more inclusive, earlier, and faster genetic diagnosis to enable tailored management.

**Table:** 

**What is Known:** *• Genetic disorders are suspected in many neonates but only genetically confirmed in a minority.* *• The presence of a genetic disorder can be easily missed and will often lead to a diagnostic odyssey requiring extensive evaluations, both clinically and genetically.*	
**What is New:** *• Different aspects of the clinical features and uptake of genetic test in a NICU cohort.* *• The need to improve genetic care in the NICU for more inclusive, earlier, and faster genetic diagnosis to enable tailored management.*

**Supplementary information:**

The online version contains supplementary material available at 10.1007/s00431-021-04213-w.

## Introduction

A significant portion of neonates admitted to neonatal intensive care units are diagnosed with a genetic disorder [1]. Congenital malformations, potentially indicating an underlying genetic disorder, are estimated to be present in 13% of all admissions to neonatal intensive care units (NICUs) and remain one of the leading causes of neonatal mortality (25–34%) [2, 3, 4, 5, 6]. The clinical presentations of genetic disorders vary widely, from an isolated (major) congenital anomaly (CA) or multiple malformations (MCA) to more subtle clinical signs or symptoms. The diagnostic pathway is often long and requires extensive evaluations that may be invasive and costly [1]. Diagnosis of most genetic disorders in neonatal and pediatric intensive care units (NICUs and PICUs) is generally not timely enough to adequately guide acute clinical management.

Previous studies have shown that genetic disorders are a frequent cause of CA, especially MCA [1, 2]. However, the exact frequency is unknown, as percentages reported vary between 20 and 50**%**, which can mainly be attributed to cohort selection and the heterogeneity of diagnostic tools used [2, 7]. In neonates admitted to a NICU, genetic testing is generally aimed at detection of aneuploidies (such as trisomy 13, 18, and 21) or chromosomal aberrations, which in lesser extent is followed by direct testing of specific genes, guided by the patients’ phenotype.

Over the last decade, novel technologies, such as whole exome sequencing (WES), have entered the genetic diagnostic arena. Its use in clinical settings, such as neonatal intensive care, have however been limited, as turnaround times were perceived too long (i.e., months) to impact acute or short-term clinical decision making, and too costly compared to other genetic diagnostic testing options [8–10]. Yet, as these turnaround times and costs have decreased significantly, there is an opportunity for innovation and durable implementation of WES in the NICU setting.

To facilitate these efforts, insight into current practices, both at the level of clinical presentation as well as the uptake of (the type of) genetic testing, is essential. For this purpose, a retrospective observational study was performed in a cohort of neonates admitted to the NICU of the Radboud university medical center during a 2-year period up to a postnatal age of 2 years.

## Methods

### Retrospective cohort definitions

We collected data of all patients born between 1 October 2013 and 1 October 2015 and admitted to the level IV NICU of the Radboud university medical center. Exclusion criteria were genetic testing in the context of a known mutation within the family and/or the identification of disorders through the national neonatal blood spot screening program [11]. For the purpose of this study, we stratified the data to three different time periods, being prenatal (before birth), neonatal (day of birth—day 1, up to 28 days of life), and post-neonatal (beyond 28 days of life). In addition, patients were categorized in six groups based on the moment when a genetic disorder was suspected (prenatal, neonatal, and post-neonatal period), combined with whether or not a genetic diagnosis was confirmed.

### Data collection and analysis

Data was extracted from the electronic medical record (EMR) for each subject until the postnatal age of 2 years. A combination of automatic and manual data extraction was performed. Information regarding demographic data, diagnoses, and clinical geneticist consultations were manually extracted from the EMR of all patients. Genetic diagnosis was defined as a molecular, cytogenetic, or metabolic abnormality explained by a genetic disorder and related to the patient’s presenting phenotype. “No genetic diagnosis” was classified as patients with (non-specific) symptoms, such as feeding difficulties or respiratory distress and physical abnormalities without confirmation of an underlying genetic disorder.

For each patient, we retrieved information on whether or not a clinical geneticist was consulted. If so, information on the date, location (inpatient /outpatient), and indication for consultation was obtained. We reviewed all genetic tests and recorded the type and result of the test, the date the specimen was received by the lab, and the date of the final report. We also included relevant tests performed prior to transfer to our institution using the information available in our EMR. Gene tests that were ordered as a panel (more than one gene), but for which results for each gene were provided separately, were entered as individual gene tests as the turnaround time may vary per gene. A conclusive diagnosis was defined as a laboratory-confirmed genetic diagnosis based on the identification of a (likely) pathogenic (classes 4 and 5) variant in concordance with the patient’s phenotype [12]. Of note, interpretation of variants also relies on the clinical presentation of the patient. Phenotypic presentation of (premature) neonates may differ from the presentation later in life for known genetic disorders [13]. The variants of unknown significance (VUS; class 3) were only considered clinically relevant if the phenotype matched appropriately as evaluated by expert clinical geneticists [14].

### Primary end points

Primary end points were (I) confirmed genetic disorders, (II) incidence of genetic testing, (III) diagnostic yield of genetic testing, and (IV) time to diagnosis (TTD). Suspicion of an underlying genetic disorder was based on the presence of one or more CA or other guiding clinical symptoms. The incidence of genetic testing was defined as the percentage of patients that received any molecular, cytogenetic, or metabolic diagnostic testing. The diagnostic yield was defined as the percentage of cases for whom a conclusive molecular or cytogenetic diagnosis was identified, e.g., the identification of a class 3, 4, or 5 variant, that is compatible with the identified phenotype. The TTD was measured from the moment the first test was indicated until the return of the final conclusive genetic diagnostic report.

### Identification and scoring of congenital anomalies

To identify the presence of any CA, we analyzed all EMR and scored the reported anomalies. Only anomalies that were identified prenatally or during the NICU stay were scored. CA were scored using the human phenotype ontology (HPO) terms and concomitantly grouped in 23 different organ systems [12]. CA were considered as isolated when affecting a single organ system, and as multiple in the presence of anomalies in two or more organ systems.

#### Statistical analysis

Normal distributed data were expressed in mean and standard deviation. Median and interquartile ranges were used in data with a skewed distribution. Statistical analysis was performed using descriptive and chi-square analyses and a two-sided Fisher’s exact test for continuous variables.

## Results

**Fig. 1:**
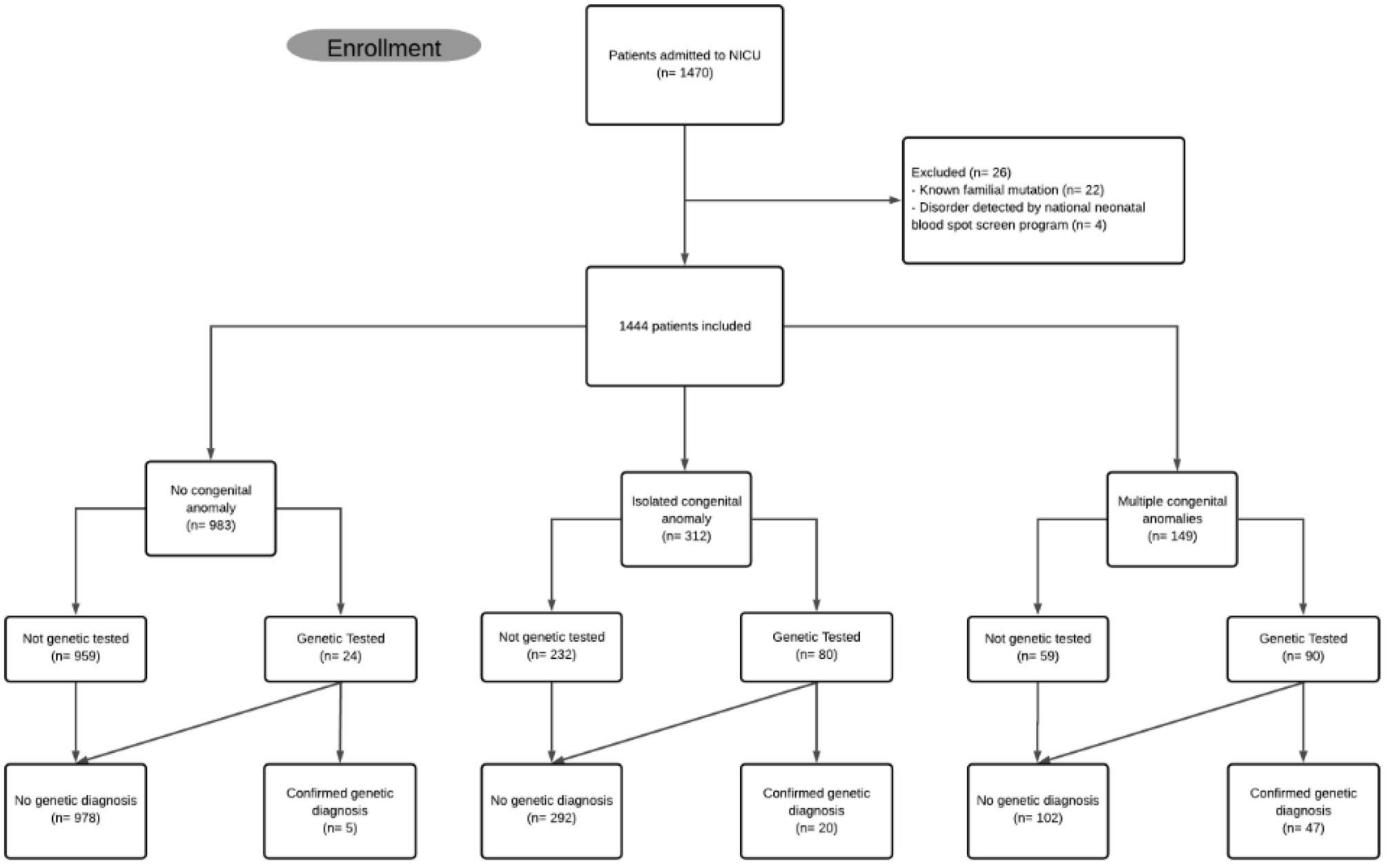
Flowchart of the study

During this 2-year timeframe, 1470 patients were admitted to the NICU; 26 patients were excluded from the analysis, because genetic testing was performed in the context of a known familial mutation (*n* = 22) or they were admitted after the identification of a neonatal bloodspot screening disorder (*n* = 4) (Fig. 1). This resulted in 1444 eligible patients. The clinical characteristics of the included patients are shown in Table 1 and Supplementary Table S1.

**Table 1:** Clinical characteristics of neonates

	*N* = 1444
Male/Female	833 (58%)/611 (42%)
Gestational age	
Extremely preterm (< 28 weeks)	91 (6%)
Very preterm (28–34 weeks)	193 (13%)
Preterm (34–37 weeks)	401 (28%)
Term (37–42 weeks)	746 (52%)
Post-term (> 42 weeks)	9 (0.6%)
Congenital anomalies	
Unknown	4 (0.3%)
No congenital anomalies	983 (68%)
Congenital anomalies	461 (32%)
• Isolated	312 (68%)
• Multiple	149 (32%)
Genetic testing	
One or more genetic tests	194 (13%)

## Genetic testing

**Fig. 2:**
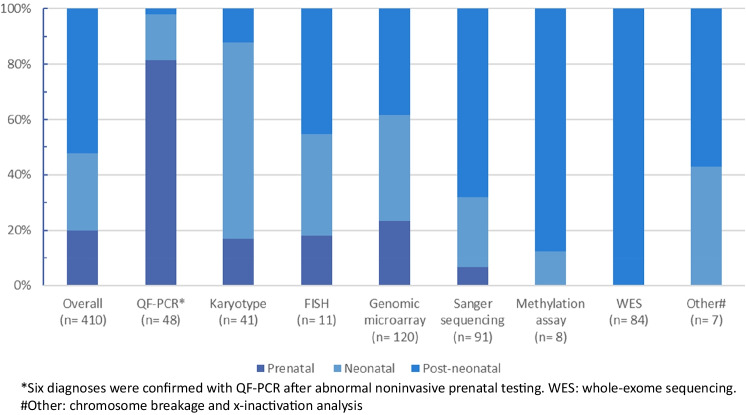
Percentage of all genetic tests per time period. QF-PCR quantitative fluorescent polymerase chain reaction

In a total of 194 patients (194/1444; 13%), 410 genetic tests were performed (Fig. 2). Of these genetic tests, 28% (114/410) were ordered in the neonatal period. More than half of the genetic tests (214/410; 52%) were initiated in the post-neonatal period. The type of genetic test varied among patients and depended on the suspected genetic disorder and corresponding clinical features. In the prenatal and neonatal period, QF-PCR, karyotyping, and genomic microarray technologies were the most frequently used diagnostic tools, whereas in the post-neonatal period, this included also Sanger sequencing and WES (Supplementary Table S2).

### Genetic diagnosis

**Fig. 3:**
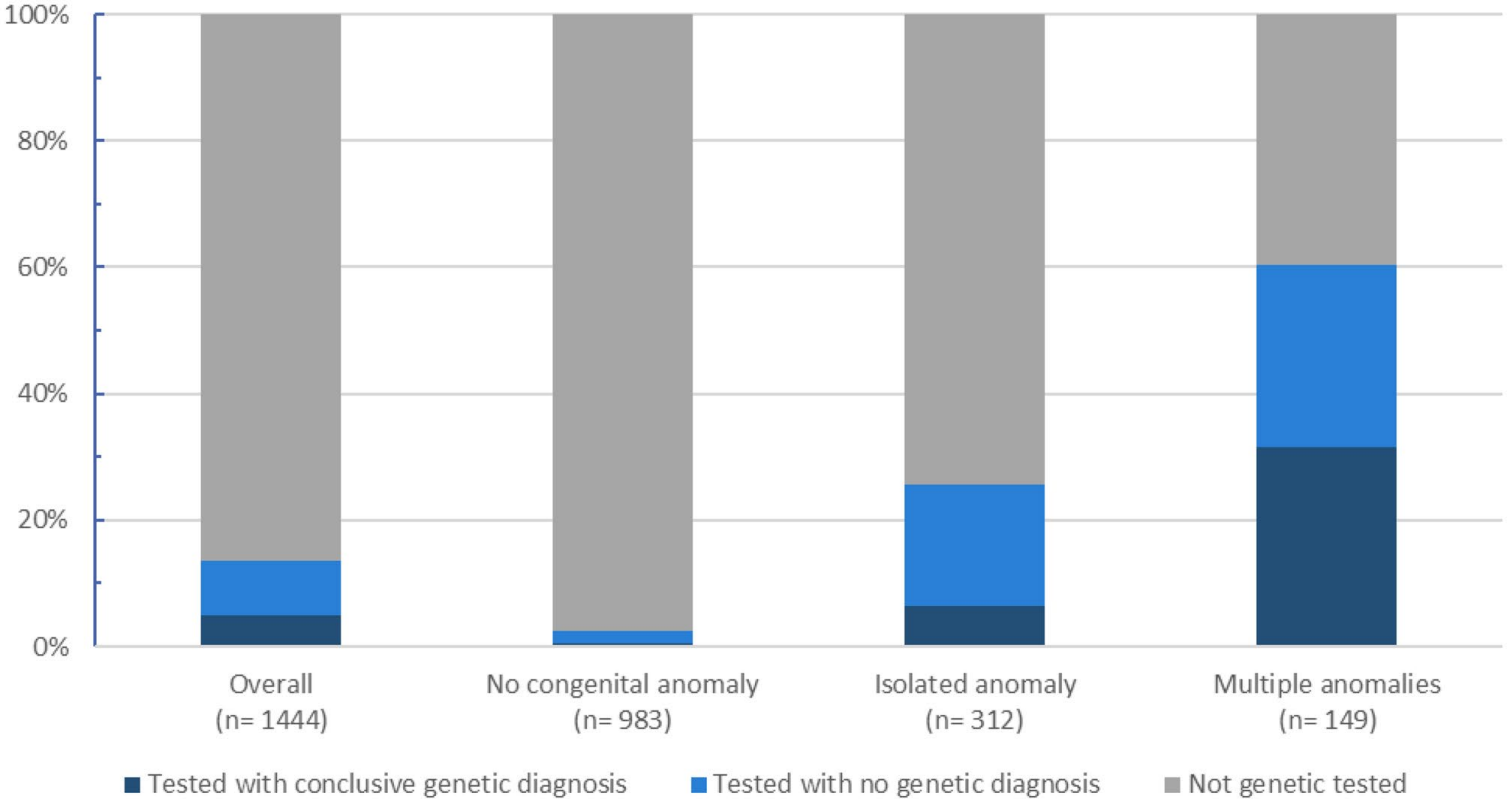
Relative frequencies for the occurrence of congenital anomalies in the total cohort in relation to genetic testing and its outcomes

In a total of 72 patients, a genetic diagnosis could be established (Table 2). The overall diagnostic yield of tested patients is 37% (72/194). We identified all genetic tests and the periods wherein these tests were performed (Supplementary Table S2). Most genetic diagnoses (38/72; 71%) were confirmed in the post-neonatal period but before 2 years of age. The timing of genetic diagnosis across the cohort is demonstrated in Tables 2 and 3. For the majority of patients receiving their genetic diagnosis in the post-neonatal period (22/38; 58%), the search for a genetic diagnosis already started in the prenatal and/or neonatal period (Fig. 3).

**Table 2:** Time to diagnosis for 72 patients with a conclusive genetic diagnosis in relation to the moment genetic testing was started, and a genetic diagnosis was confirmed.

	Prenatally confirmed diagnosis	Neonatally confirmed diagnosis	Post-neonatally confirmed diagnosis
Suspected genetic disorder prenatally (*n* = 21)	15	1	5
Suspected genetic disorder neonatally (*n* = 35)	n/a	18	17
Suspected genetic disorder post-neonatally (*n* = 16)	n/a	n/a	16

*n/a* not applicable

**Table 3:** Time period in which genetic testing started and the timing of genetically confirmed diagnosis

	Prenatally confirmed diagnosis (*n* = 15)	Neonatally confirmed diagnosis (*n* = 19	Post-neonatally confirmed diagnosis (*n* = 38)
Median time (Q1–Q3) to diagnosis (days)	n/a	22 (5–12)	112 (33–268)
Median age (Q1–Q3) start genetic testing (days)	Prenatally	2 (1–6)	17 (5–309)
Median postnatal age (Q1–Q3) at genetic diagnosis (days)	Prenatally	13 (6–20)	209 (67–550)
Median number of genetic tests	2	1	2

*n/a* not applicable, *Q1* first quartile, *Q3* third quartile

The median time to diagnosis for patients with a confirmed genetic diagnosis in the post-neonatal period was 112 days (IQR 234 days). Patients in the post-neonatal period received more genetic tests than patients in the neonatal period (Table 3). The type of genetic tests most used in the post-neonatal period often has a long turnaround time. These factors have a significant impact on the median time to diagnosis for the patients.

### Congenital anomalies

**Fig. 4:**
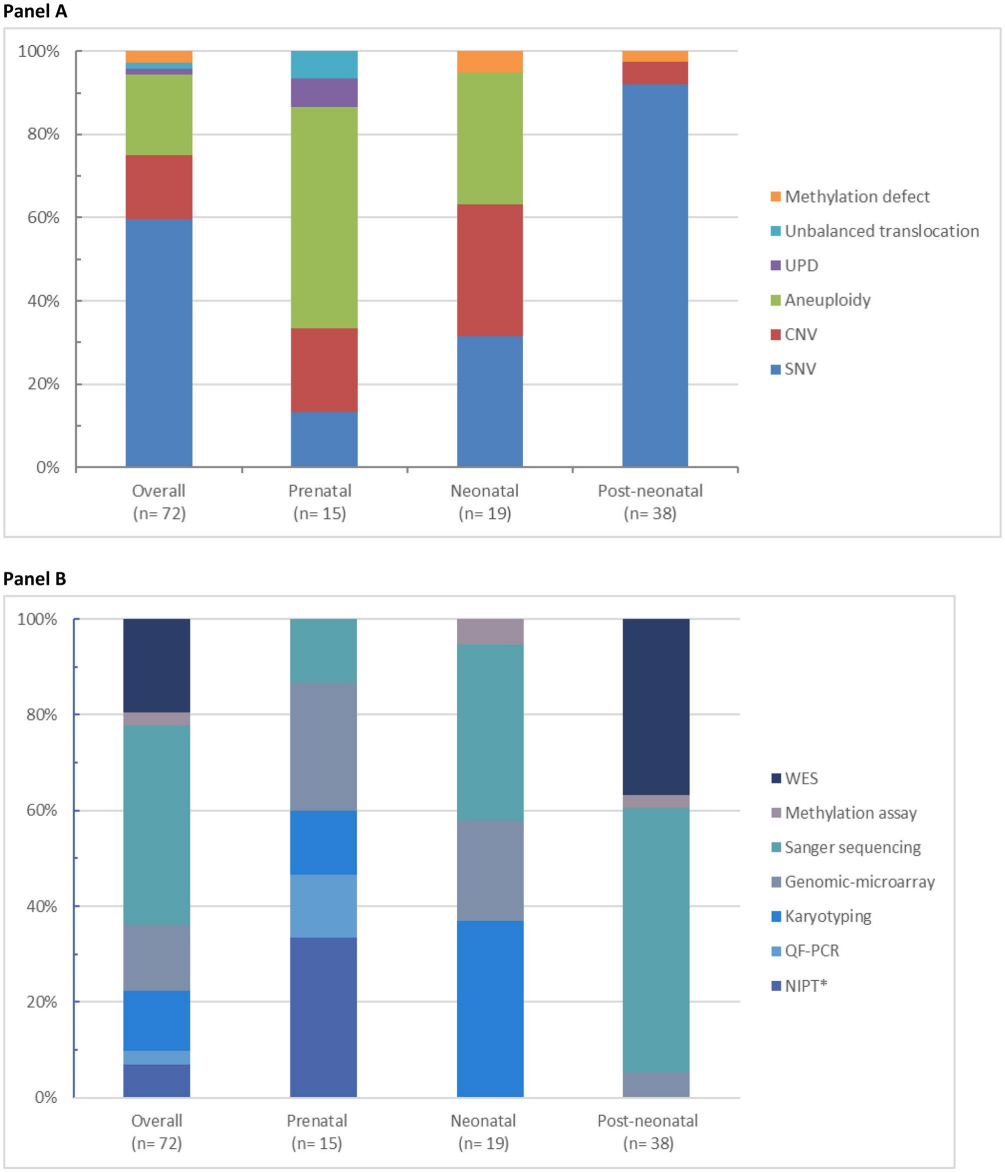
Relative contribution of genetic assay establishing the conclusive genetic diagnosis in relation to moment of testing (panel **A**) and type of genetic alterations identified (panel **B**). Panel **A** UPD uniparental disomy. CNV copy number variant. SNV single-nucleotide variant. Panel **B** NIPT noninvasive prenatal testing. *Six abnormal NIPTs were confirmed with QF-PCR. QF-PCR quantitative fluorescent polymerase chain reaction. WES whole-exome sequencing

CA were identified during the NICU admission in 32% (461/1,444) of patients, of whom 68% (*n* = 312) presented with an isolated CA and 32% with MCA (*n* = 149; Table 1). Uptake of genetic testing correlated with the categories for CA: 24/983 (2%) of patients without CA were tested, 80/312 (26%) of patients with an isolated CA, and 90/149 (60%) of patients with MCA. As expected, also, the diagnostic yield correlated with these groups, with 21% (5/24) obtained in patients without CA, 25% (20/80) for those with an isolated CA, and 52% (47/90) for patients tested with MCA (Fig. 4). In reverse, patients with a CA represented 67/72 (93%) of the confirmed genetic diagnoses. Approximately two-thirds (44/67) of the diagnosed patients had MCA. This group of patients with MCA will be most of the time tested independently of the affected organ systems. Of note, there was no difference in the frequency of affected organ system between diagnosed and undiagnosed patients with an isolated CA nor with the uptake of genetic testing.

### Types of genetic defects

An overview of types of genetic defects is displayed in Fig. 3, and details for all genetic disorders are presented in supplementary Table S3. Overall, more than half (43/72; 60%) of the detected genetic defects were single-nucleotide variants (SNVs), responsible for monogenic disorders with large genetic and clinical heterogeneity. These genetic disorders were predominantly (35/43; 81%) diagnosed in the post-neonatal period, by unbiased genome wide technologies such as exome sequencing and/or gene panel-based strategies. Fifteen out of 72 (21%) neonates had an aneuploidy, which were detected prenatally or neonatally by use of technologies such as karyotyping and QF-PCR. Of the latter, the commonly identified genetic disorders were Down’s syndrome/trisomy 21 (10/72; 14%), Patau’s syndrome/trisomy 13 (1/72; 1%), Edward’s syndrome/trisomy 18 (2/72; 3%), and Turner syndrome (2/72; 3%). The remaining 19% (14/72) of genetic defects (copy number variants (CNVs), uniparental disomy, and methylation defect) were predominantly detected by genomic microarray and methylation assay.

## Discussion

In this study, we retrospectively evaluated a cohort of 1444 neonates admitted to the NICU for the presence of genetic disorders and the genetic diagnostic process during the first 2 years of life. We observed that approximately one-third of all neonates at the NICU present with CA, which is often an indication for genetic testing. However, the timeline of genetic testing as part of the diagnostic pathway usually exceeds the neonatal time period. Also, the genetic technologies used during the neonatal period differ from those used in the post-neonatal time frame. In the last years, advanced techniques, with higher diagnostic yields, like exome or genome sequencing are used to diagnose patients in the post-neonatal period.

Neonates with congenital malformations indicating a possible genetic disorder comprise a substantial proportion of NICU admissions. Congenital malformations are important signs and should always alert the clinician [3, 7]. The presence of a genetic disorder can easily be missed because of the variable clinical presentation of genetic disorders, often leading to a diagnostic odyssey requiring extensive evaluations, both clinically and genetically [1].

Identifying the genetic cause of a patient’s condition puts an end to the diagnostic odyssey, obviating the need for further costly testing. Furthermore, confirmation of a genetic diagnosis has also been shown to alter clinical management [8, 10, 15–17]. This may lead to a reduction in mortality and morbidity related to genetic disorders with onset in newborns. Contrarily, it may facilitate shared decision-making regarding transition to palliative care [8, 18–20]. As an example, in one patient with congenital alveolar capillary dysplasia with misalignment of the pulmonary veins and therapy-resistant pulmonary hypertension, the TTD extended beyond 1 month. Prolonged ineffective cardiorespiratory support could be prevented for this patient given the disastrous prognosis due to the underlying genetic disorder. The timing of diagnosis may have major impact on clinical management of critically ill neonates [17].

Interestingly, we noted that patients suspected of a genetic disorder in the neonatal period were more likely to get a diagnosis faster compared to a resulting suspicion in the post-neonatal phase. The median TTD of patients tested in the prenatal or neonatal period was significantly shorter compared to patients tested in the post-neonatal period. The reasons for this are the shorter turnaround time of the genetic tests used in these patients compared to those who are tested later in life, but also more obvious clinical presentations, like major CA, in neonates which initiated genetic testing compared to for instance developmental disorders or isolated intellectual disability, which only become recognizable later in life [21].

In this study, we identified all patients with CA and determined their genetic diagnostic path throughout their first 2 years of life. It was observed that 26% (80/312) of infants with an isolated CA were genetically tested, leading to a diagnosis in 25% (20/80) of these patients. Similarly, for patients with MCA, 60% (90/149) of patients received genetic testing, with a diagnostic yield of 52% (47/90). Comparison of the different clinical presentations to determine whether we could identify any clinical indications why some patients with CA were tested, and others were not, did not reveal any specific observations (data not shown). This was not dependent on which organ system was affected. Potential reasons for the reduced uptake of genetic testing in patients with isolated CA or MCA could be unawareness of physicians to order genetic testing, or perceptions of “too long turn-around times to impact clinical decision making” and/or parents rejecting genetic evaluation.

Following the above rationale, one may wonder how many patients with CA would have benefited from early genetic testing, thereby reducing their diagnostic odyssey and allowing enhanced patient-tailored medicine. Extrapolation of the data from our cohort and based on the assumption that the genetic diagnostic yield achieved is representative for the remainder of the cohort, an extra 58 (diagnostic yield of 25% in 232 not-tested patients) patients could potentially be diagnosed in the group with an isolated CA, and another 25 (diagnostic yield of 43% in 59 not-tested patients) patients in the sub-cohort with MCA. The diagnostic yield for the group with MCA is corrected for patients with aneuploidies who rarely will not be tested and diagnosed because of the obvious clinical features.

Of note, also in the group of patients without CA, genetic diagnoses were made; retrospective analysis of these patients showed that there were specific clinical indications (mostly later in life) for genetic testing, such as neurodevelopmental delay. As we have limited the follow-up period of our cohort to 2 years of age, it is currently not possible to extrapolate the potential for additional diagnosis in the group of patients without CA. Overall, it is speculated that at least 83 (58 plus 25 = 6%) patients in our total cohort of 1444, and 29% (83/291) of not-tested patients with a CA could have likely remained undiagnosed due to a lack in genetic testing.

The limited uptake of genetic testing in daily clinical practice on the NICU patients offers opportunities for improvement, for instance, by offering genetic testing to all patients with one or multiple CA. Traditionally, genetic testing has been too time-consuming or perceived to have limited impact on management of the critically ill neonate. Technological advances in recent years have led to the ability to sequence and interpret the entire genome of a neonate in only 1 or 2 days [22, 23]. Whereas many others have already shown that exome or genome sequencing can effectively be used to diagnose patients in turnaround times required in an acute setting, other clinical utility questions remain unsolved [1, 9, 17–20, 23–26]. This does not only include matters related to genetic consultation, and patient selection, but also socio-economic analyses on cost-effectiveness, and scenario models to determine the most effective strategy to test most, if not all, patients at the NICU.

Ideally, one would analyze these aspects in a prospective parallel study that would offer great insight into the opportunities and potential pitfall of a so-called “WES or WGS-first strategy.” Outcome measures should not only focus on quantification of the diagnostic yields via rapid WES, but also on relevant clinical management changes, which are anticipated to range from the initiation of specific patient-tailored supportive management, the transition to palliative care for confirmed lethal conditions to simply refraining from further invasive diagnostic procedures as a consequence of having a final molecular diagnosis [8]. The parental perceptions of WES are also very important, and key factors in the process of empowerment must be explored.

Whereas our study has limitations because of its retrospective nature relying only on information available in the patients’ EMR with only a few years of clinical follow-up, its power is reflected by the systematically assessment of all patients admitted to a level IV NICU for their clinical presentation, genetic testing, and genetic diagnosis obtained. We have motivated the speculation that currently admitted neonates to the NICU are underdiagnosed for disorders of genetic origin. Our results contribute to gaining insight in patient populations that would benefit from WES- or WGS-based genetic testing, that not only allows for impact on clinical decision making in the acute setting but would also limit the diagnostic odyssey of these patients. However, further research is needed to determine the best strategy on whom to offer advanced genetic testing, maximizing the potential of WES or WGS in the NICU setting improving the care provided to infants and their families.

## Supplementary information

Below is the link to the electronic supplementary material.

Supplementary file1 (XLSX 92 KB)

Supplementary file2 (XLSX 27 KB)

Supplementary file3 (XLSX 28 KB)

## Abbreviations

CA: Congenital anomaly
MCA: Multiple congenital anomalies
NICU: Neonatal intensive care unit
WES: Whole-exome sequencing
